# Prospective observational studies on nutrition intake and the incidence of cognitive impairment in middle-aged and older adults: A protocol for systematic review and meta-analysis

**DOI:** 10.1371/journal.pone.0287852

**Published:** 2023-06-29

**Authors:** Hailun Jiang, Weiming Zhu, Boxuan Li, Shizhe Deng, Xianggang Meng, Wei Liu, Yuzheng Du, Zhihong Meng

**Affiliations:** 1 Department of acupuncture, First Teaching Hospital of Tianjin University of Traditional Chinese Medicine, Tianjin, China; 2 Graduate School, Tianjin University of Traditional Chinese Medicine, Tianjin, China; 3 Department of acupuncture, National Clinical Research Center for Chinese Medicine Acupuncture and Moxibustion, Tianjin, China; University of Liege: Universite de Liege, BELGIUM

## Abstract

**Introduction:**

According to several studies, a specific dietary pattern can reduce the risk of dementia and cognitive impairment. However, the robustness of these results has not been tested. The study intends to investigate the association between nutrition intake and cognitive impairment in middle-aged and older adults (≥45-years) and provide reliable, evidence-based references for healthcare decision-makers, researchers, and policymakers.

**Review question:**

Are the dietary characteristics of community-dwelling adults (≥45-years) associated with the occurrence of cognitive impairment?

**Objectives:**

The primary objective of this protocol is to synthesize the longitudinal observational evidence on the relationship between nutrition intake patterns and the incidence of cognitive impairment in middle-aged and older adults (≥45-years), and to provide detailed dietary recommendations for the prevention of cognitive impairment in this population.

**Methods and analysis:**

Cohort studies conducted among adults (≥45-years) will be included. The following electronic databases will be searched for relevant records published by July 2023, with a restriction on language to English: Pubmed, Medline, Embase, Web of science, Cochrane Library. The studies will be selected, the data will be extracted, and the bias risk will be assessed by two independent investigators. The Meta-analysis of Observational Studies in Epidemiology guidelines will be followed to summarize observational studies, and the protocol will adhere to the recommendations from the Preferred Reporting Items for Systematic Review and Meta-Analysis Protocols 2015 statement. Endnote X9 will be used to manage data screening. We will use Review Manager 5.4 and Stata 16.0 to conduct data analysis, and a random-effects model will be applied to pool clinically homogenous studies. The results will be presented based on the form of nutrition intake. For assessing publication bias, Egger’s test and visual inspection of funnel plots will be utilized.

**Ethics and dissemination:**

As this study does not involve primary data, ethical approval is not required. The final report will be published in a peer-reviewed journal.

**Prospero registration number:**

A registration number of DOI 10.17605/OSF.IO/NAKC3 was assigned to it on October 15, 2022 on Prospero.

## Introduction

Mild cognitive impairment (MCI) is characterized by a decline in cognitive performance which exceeds age and education expectations, and impairment in one or more cognitive domains, while functional abilities remain independent. This is distinct from dementia, which is characterized by more severe cognitive deficits and evidence of significant impairment in social or occupational functioning [1]. In October 2021, The Global Dementia Status Report from the World Health Organization (WHO) provided new estimates on the burden and cost of dementia. The report indicated that there were 55.2 million people worldwide living with dementia in 2019, which cost 1.3 trillion USD. Furthermore, dementia has become the 7th leading cause of death. Mild cognitive impairment is a transitional state between typical cognitive aging and early dementia, and is considered to be the preclinical stage of dementia [2]. As a result, the number of individuals with cognitive impairment who have not yet progressed to dementia is expected to be even higher [3]. One study predicted the global prevalence of MCI among community dwellers is over 15% [4]. Fortunately, an increasing number of countries have recognized the growing problem of dementia as a global public health challenge and have developed corresponding national policies or plans (https://www.who.int/). Prevention, delay, and mitigation the effects of dementia are currently the focus of research and guideline development efforts.

The European Society for Clinical Nutrition and Metabolism (ESPEN) recommends oral nutrient supplementation (ONS) only as a last resort for the prevention or correction of cognitive decline [5]. The Chinese Society for Parenteral and Enteral Nutrition (CSPEN) recommends developing healthy habits and maintaining a balanced nutrition to prevent or delay the occurrence of cognitive impairment [6]. One hypothesis proposes that the impact of nutrition on cognitive health may be influenced through various pathways, such as inflammatory, immune, or cholesterol metabolic pathways [7, 8]. Of these pathways, neuroinflammation, which can be induced by oxidative stress responses, has the potential to cause neural degeneration or neuronal death within the brain, ultimately leading to memory loss [9]. Furthermore, a scientific statement from the American Heart Association (AHA) suggests that neuroinflammation and systemic inflammation may be factors in the association between various underlying medical conditions (such as stroke, diabetes, and sleep apnea) and cognitive decline in older adults [10]. Evidence suggests that adhering to the Mediterranean dietary pattern, which emphasizes a diet rich in fruits, vegetables, legumes and unrefined grains, moderate dairy products, and low in meat but regular intake of fish, may support maintenance of cognitive function and reduce the risk of cognitive decline in healthy older adults by providing sufficient amounts of all nutrients [11]. The European Food Safety Authority’s (EFSA) Scientific Committee has issued a Guidance on the human health risk-benefit assessment of foods, considering both the human health risks and human health benefits associated with food consumption. According to the guidance, consuming oily fish at least once a week, which are rich in n3-PUFAs, can have positive effects on cognitive health [12]. In addition, Dietary approaches to Stop Hypertension (DASH) diet, the Mediterranean-DASH diet Intervention for Neurodegenerative Delay (MIND) diet and anti-inflammatory diet all appear to have positive effects on cognitive health outcomes in older adults [13]. But, balancing nutrition remains a question. The 2022 ESPEN micronutrient guideline emphasizes the importance of considering the timing of treatment and timely measurement when administering micronutrients (MNs), and recommends strict control of the tolerable upper intake levels to prevent the risk of overdose, toxicity, or serious side effects [14]. Moreover, restrictions on dietary intake for older adults, such as low sugar for diabetics, low salt for hypertensive patients, and low cholesterol for hypercholesterolemia, appear to be less effective with regard to relevant study endpoints [15]. And according to a position statement from the American Dietetic Association(ADA), to enhance the nutritional intake and improve the quality of life for older adults, restrictions on dietary intake should be eased. This approach may aid in mitigating the potential increased risk of dementia and cognitive impairment associated with malnutrition [16]. However, it is noteworthy that the recommendation’s level of evidence is of very low, as rated by the ESPEN [5].Therefore, there is an urgent need for comprehensive evidence to explore the relationship between nutritional intake patterns and the incidence of cognitive impairment.

As it is widely acknowledged that cognitive impairment impacts dietary habits and leads to reduced diet variety and imbalanced nutrient intake [5, 17], in turn, certain dietary patterns may alleviate the risk of dementia and cognitive impairment [18–20]. However, cross-sectional evidence alone may not be sufficient to establish a causal relationship between diet and cognitive impairment. Longitudinal observational evidence conforms to the chronological order of causality, and can objectively reveal causation between cognitive impairment and nutrition intake.

There are few intervention studies on the impact of dietary intervention on cognition in older adult, and they typically involve participants who already exhibit cognitive impairment [21]. The limited number of randomized controlled trial makes it difficult to verify the efficacy of dietary interventions [22, 23], as such trials are often constrained by the study period, patient compliance, funding, and manpower. Therefore, a prospective cohort study on a large population can avoid the causal effect of cross-sectional study on the one hand, and save significant amounts manpower and financial resources on the other hand.

A systematic review has shown that the effects of dietary interventions on cognitive ability are more likely to be detected in older participants(≥65-years) [24], considering that is relevant to the incidence of dementia is steep increase with age [25]. Moreover, dementia has the characteristics of being clinically silent in middle-aged populations with accumulating the pathological hallmarks decades before the onset of clinical symptoms [11, 26]. After reviewing the literature, the Scientific Advisory Group of the European Prevention of Alzheimer’s Dementia (EPAD) summarized studies that focused on the correlation between cognitive function and changes in preclinical brain imaging, as well as cohort studies that examined the development of incident dementia in non-dementia populations. Based on their findings, the group arrived at a consensus that early intervention during the preclinical stage of dementia may provide a more effective intervention window [21]. Implementing dietary preventive intervention strategies in early old age may reduce the incidence of cognitive dysfunction in the total population. Hence, finding the optimal time window for dietary intervention is crucial in altering the course of the disease. We select the middle-aged and older adults (≥45-years) as our study population.

Cognitive decline is an inevitable aspect of natural aging, yet there are noteworthy inter-individual differences. These differences could arise from lifestyle factors, including nutritional intake. Furthermore, several systematic reviews have investigated the association between nutritional intake and cognitive impairment [13, 27, 28]. These systematic reviews analyzing the current evidence on the correlation between nutrient intake and cognitive outcomes among middle-aged and older adults deduce that modifying diet could serve as a preventative strategy against cognitive decline in this population is feasible and may mitigate its onset. Meanwhile, the studies included in these systematic reviews are potential candidates for meeting the inclusion and exclusion criteria for our study. These systematic reviews provide analyzable data, but these reviews are insufficient as they did not undergo meta-analysis. Therefore, further exploration of this information is necessary. The incidence of dementia or cognitive impairment will serve as the outcome indicator for our data analysis. The previous systematic reviews lacked a combined analysis of data from multiple cohort studies. We will comprehensively analyze the data to gain a better understanding of the relationship between cognitive decline and dietary patterns. Besides, in order to reduce heterogeneity among clinical studies, this community-based and person-centred meta-analysis will only focus on longitudinal cohort studies. Perhaps, modifying dietary patterns may be a potential option for preventing the risk of cognitive impairment and dementia in both clinical practice and public health settings.

## Methods and analysis

Preferred Reporting Items for Systematic Reviews and Meta-Analysis protocols (PRISMA-P) was used to develop a systematic review and meta-analysis protocol and PRISMA-P guidelines will be used for reporting the entire manuscript [29]. The PRISMA-P checklist is attached as S1 File.

## Inclusion and exclusion criteria

### Include

#### 1. Population

Community-dwelling participants aged 45 years and older (≥45-years) at baseline, or participants were identified as subgroups of 45 years and older (≥45-years) at baseline in the study

#### 2. Exposures

Focus on the content of the food including any form of nutrition intake (e.g. dietary patterns, food groups, foods and nutritional supplements (e.g.Vitamins, minerals and protien)).

Note: There may be heterogeneity in dietary patterns among studies included in this analysis. Therefore, we will use the definitions of dietary patterns as reported in the original literature and will not categorize the diet types in this study.

#### 3. Outcome

Incidence of dementia or cognitive impairment in this population.

#### 4. Study design

Prospective observational cohort study.

### Exclude

#### 1. Population

(1) Participants with specific medical conditions (e.g. Diabetes mellitus, psychiatric disorders, HIV infection, substance abuse, liver diseases, cardiovascular/cerebrovascular disorders, and overweight/obese populations)

(2) Participants already had cognitive impairment at the time of cohort entry

#### 2. Exposures

(1) Examining only dietary habits (e.g. fasting or binge eating)

(2) Examined only biomarkers of nurtrition (e.g. homocysteine or carotenoids in plasma [30])

#### 3. Outcome

(1) Examining only self-reporting

#### 4. Study design

Non-original data such as protocols, letter, comments, etc.

#### 5. Language of study

Non-English-written studies.

## Search strategy

We will conduct a search of several electronic databases, including Pubmed, Medline, Embase, Web of Science, and Cochrane Library. There are several key topics covered in the title/abstract, including nutrition, cognitive, dementia, aging, incidence, and cohort. A list of related terms is provided in S1 Table. A tentative search was conducted on April 23, 2023 and 2426 articles were retrieved in the Pubmed and search term is recorded in the S2 File. In addition to our traditional database searches, we will also perform a search of grey literature using Google Scholar. Furthermore, our next step will be to scan the references list of eligible articles, in order to identify any grey literature that may not have been covered in our initial search. To obtain complete data for eligible studies, we will reach out to key authors and request any pertinent information, including supplementary materials that may not have been fully published or reported and information from informal sources of relevant research. The anticipated start date is July 2023.

## Selection of studies for inclusion in the review

**Fig 1:**
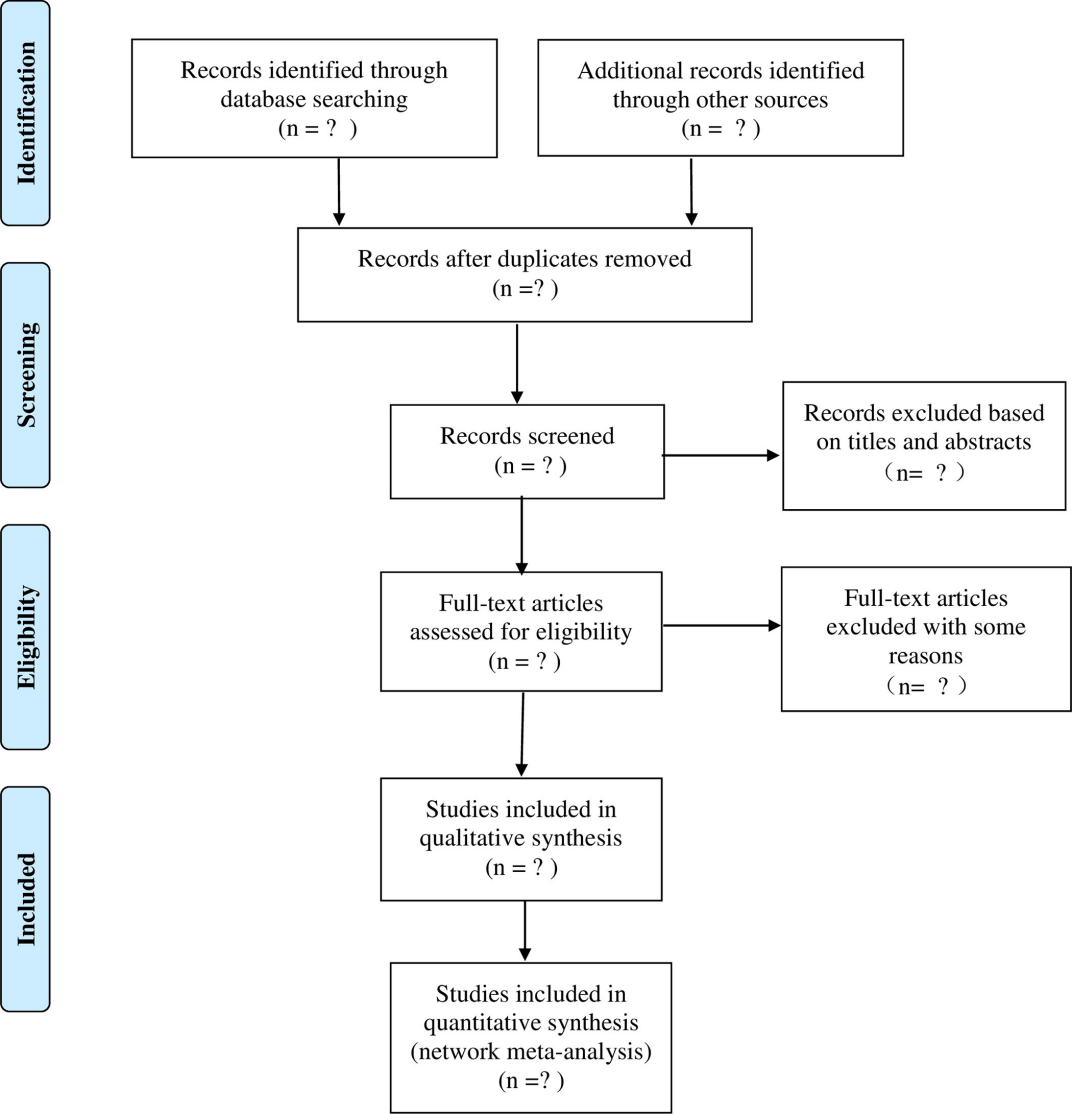
Flow chart for identifying and selecting studies.

All preliminarily eligible articles will be uploaded to Endnote X9 and articles’ titles and abstracts obtained from the electronic databases will be scanned by two independent investigators (JHL and ZWM). Then two investigators will review the full texts to select the articles that meet the inclusion criteria, keeping a separate record of reasons for article rejection. Disagreements will be resolved through discussion, and if disagreements persisted, then the third investigator (LBX) was consulted until consensus is reached. The resulting articles that meet the requirements will be summarized. As shown in Fig 1, the selection process follows a flow chart.

## Methodological quality assessment and data reporting

The quality of longitudinal observational studies will be assessed independently by JHL and ZWM through the Newcastle-Ottawa Quality Assessment scale (NOS) of Cohort Studies, which contains three dimensions (selection domain, comparability domain and outcome domain) including 8 items totally and the maximum score is 9 stars [31]. Two investigators will grade methodological quality of each included study into following three levels independently: good quality (8~9 stars), fair quality (5~7 stars) and poor quality (0~4 stars). In the event of any disagreement, the investigators will resolve the issue through discussion. In the case of persistent disagreements, the third investigator (LBX) was consulted until consensus was reached.

### Extraction and management of data

Data extraction forms will collect information including five domains, the characteristics of study is attached as S2 Table:

Publication information: first author, year of publication,

Participants characteristics: country, sample size, sex, age, ethnicity, educational attainment,

Exposure: identification and definition of exposed and unexposed factor, dietary pattern (type, assessment tool),

Study characteristics: follow-up period, analysis method, confounders, industry funding,

Outcomes: diagnosis of cognitive impairment (assessment tool, threshold), incidence, the result of every study.

Initially, the first investigator (JHL) will extract the data, followed by the second investigator (ZWM)’s verification, and disagreements are resolved through discussion. If disagreements persist, then the third investigator (LBX) will be consulted until the agreement is reached.

The data will be extracted from the analysis using the strictest definition of cognitive impairment when a study runs multiple analyses (due to the diversity of assessment tools for cognitive impairment, we will extract the data with the highest incidence in each study), the largest sample size, the longest duration of follow-up and covariates with the highest number.

Whenever outcome data is missing, a qualitative analysis of the findings will be provided within the narrative description. Where information is incomplete, additional information should be obtained by contacting corresponding authors via call or email.

In order to deal with the potential overlap of samples among studies, we will verify the original authors, the registration of the project, as well as the region where the project was undertaken.

### Data synthesis and analysis

When the basal dietary intake is sufficiently similar between studies, we will merge them. We will compare the highest intake versus the lowest intake of any form of nutrition, and use the pooled effect size (pooled odds ratio (OR) and its corresponding 95% confidence interval (95% CI)) to measure the association between nutrition and cognitive impairment risk. The OR value after adjusting for confounding factors as effect size removes the causal assumption restriction that risk ratio (RR) has, making it more widely used. When the incidence of cognitive impairment is low (<10%), the RR can be treated as OR [32]. Based on the RR value and the incidence of cognitive impairment (P_0_), the OR value can be calculated in the non-exposed group (lowest intake group) with the correction formula: RR = OR/((1-P_0_) + (P_0_*OR)), when the incidence of cognitive impairment is > 10%. As a result, hazard ratios (HRs) will be viewed as OR and sensitivity analysis will be conducted to determine whether excluding these studies would affect overall results [33]. Based upon the regression coefficients (β) and standard error (SE), the odds ratio (OR) and 95% CI can be calculated and the formula is OR = e^β^, 95%CI = e^(β±1.96*SE)^.

Random-effects models will be applied. I^2^ or Chi-square test p will be used to quantitatively determine heterogeneity. When I^2^<50% or Chi-square test p≥0.1, the heterogeneity is considered unapparent. Conversely, significant heterogeneity is present when I^2^≥50% or Chi-square test p<0.1 [34]. To check for publication bias, we will perform Egger’s test, and if p> 0.05, there will be considered no publication bias. In addition to Egger’s test, a funnel plot will be visually inspected to assess of publication bias. We plan to use plan a trim and fill analysis to correct for publication bias if present, which involves removing studies that appear to be outliers and imputing hypothetical “missing” studies to create a symmetrical funnel plot.

In order to determine the effect of these variables on the outcome of interest, we will perform sensitivity analysis on the results of the methodological quality assessment and primitive data type. Specifically, we will exclude studies rated as poor quality (0~4 stars) and those that only report HR. This approach will allow us to assess the robustness of our results and identify any potential sources of bias. Furthermore, we will establish subgroups analysis based on country type (high-, low-, or middle-income) and age groups (Age 45–59, Age 60–74, Age 75–89, Age ≥90 years).

### Presentation and reporting of results

Review Manager 5.4 and Stata 16.0 software will be used for data analysis. These datasets will be included in the meta-analysis when the item contains two or more datasets. However, If there are studies meet the inclusion criteria but cannot be combined with other studies for pooled analysis, we will describe the characteristics and results of the study in narrative format in the article.

## Discussion

Based on what we know, this will be the first and most comprehensive quantitative review to investigate the relationship between cognitive decline and nutrient intake in middle-aged and older adults (≥45-years). This study aims to investigate the relationship between nutrition intake and incidence of cognitive impairment, rather than the development or evolution of cognitive impairments. It is important to note that it is inevitable that the study has some limitations, and we will try our best to rectify them. First, the language restricts to English and the type of observational studies included restricts to prospective cohort studies, which limits the number of studies available for analysis. To cope with this situation, we will conduct manual retrievals in addition to searching the four major electronic databases. Another potential limitation may be that some studies are not available for meta-analysis because of lack of homogeneity. In such cases, systematic summaries will be written in narrative form. Despite these limitations, the study also has several strengths. Firstly, only cohort studies will be included to minimize reverse causality by avoiding cross-sectional studies. Additionally, only participants without cognitive impairment at baseline will be included in this study, which ensures baseline stability and reduces clinical heterogeneity.

Hopefully, this study will develop dietary recommendations for preventing cognitive impairment in middle-aged and elderly adults. Furthermore, it seeks to provide longitudinal observational evidence to support those recommendations.

## Supporting information

S1 Table Search strategy. (DOCX)

S2 Table Characteristics of study. (DOCX)

S1 File PRISMA-P-checklist. (DOC)

S2 File PubMed search history. (CSV)
