# Supplementary material for: Prospective observational studies on nutrition intake and the incidence of cognitive impairment in middle-aged and older adults: A protocol for systematic review and meta-analysis
Source: PLoS One. 2023 Jun 29;18(6):e0287852. doi: 10.1371/journal.pone.0287852 (PMC10309612; doi:10.1371/journal.pone.0287852)
Supplement: S1 Table — (DOCX) [file pone.0287852.s001.docx]

**Supplemental Table 1**

Details of search strategy in Pubmed

| ＃1 | [Mesh]:Diet, Food, and Nutrition OR Beverages OR Alcoholic Beverages OR Artificially Sweetened Beverages OR Carbonated Beverages OR Coffee OR Drinking Water OR Energy Drinks OR Fermented Beverages OR Fruit and Vegetable Juices OR Milk OR Milk Substitutes OR Sugar-Sweetened Beverages OR Tea OR Teas, Herbal OR Teas, Medicinal OR Fermented Foods OR Cultured Milk Products OR Fermented Beverages OR Silage OR Soy Foods OR Food OR Bread OR Candy OR Chocolate OR Condiments OR Crops, Agricultural OR Dairy Products OR Dietary Advanced Glycation End Products OR Dietary Carbohydrates OR Dietary Fats OR Dietary Fiber OR Dietary Proteins OR Dietary Supplements OR Edible Insects OR Eggs OR Fast Foods OR Flour OR Food Ingredients OR Food, Fortified OR Food, Genetically Modified OR Food, Organic OR Food, Preserved OR Food, Processed OR Foods, Specialized OR Fruit OR Functional Food OR Honey OR Meals OR Meat OR Molasses OR Nutrients OR Nuts OR Raw Foods OR Salads OR Seeds OR Vegetables |
| --- | --- |
| ＃2 | [Title/Abstract]:nutrition OR food* OR diet* OR nutrient* OR fruit* OR vegetable* OR grain* OR high-sugar OR high-fat OR ultra-processed OR Mediterranean* OR saturated fat OR saturated-fat OR vitamin* OR micronutrient* OR beer* OR wine* OR beverage* OR Coffee OR juice* OR milk OR tea OR Natto OR Tofu OR Bread* OR cand* OR confection* OR chocolate* OR dairy product* OR dietary fat OR dietary maillard reaction product* OR dietary AGEs OR dietary advanced glycation endproduct* OR dietary fiber* OR wheat bran* OR roughage* OR dietary Carbohydrate OR dietary protein OR dietary supplement OR dietary supplementation* OR nutraceutical* OR nutriceutical* OR neutraceutical* OR herbal supplement* OR egg OR ready to eat meal* OR flour* OR rice OR honey* OR molasse* OR meat* OR nut |
| ＃3 | ＃1 OR＃2 |
| ＃4 | [Mesh]:cognitive dysfunction OR dementia OR neurocognitive disorders |
| ＃5 | [Title/Abstract]:cognitive dysfunction* OR cognitive impairment* OR cognitive disorder* OR cognitive decline* OR dementia* OR amentia* OR amnestic OR mild neurocognitive disorder* OR cognition* |
| ＃6 | ＃4 OR＃5 |
| ＃7 | [Mesh]: Aged OR Middle Aged |
| ＃8 | [Title/Abstract]: older OR elderly OR oldest OR elder OR old OR ageing OR aging OR post-menopausal* OR postmenopausal* OR aged OR middle age |
| ＃9 | ＃7 OR＃8 |
| ＃10 | [Title/Abstract]: incidence OR incident OR new Cogniti* OR subsequent Cogniti* OR new case OR onset OR risk |
| ＃11 | [Mesh]: Cohort Studies |
| ＃12 | [Title/Abstract]:cohort OR prospective* OR longitudinal OR follow-up OR follow up |
| ＃13 | ＃11 OR＃12 |
| ＃14 | ＃3AND＃6AND＃9AND＃10AND＃13 |
