# Supplementary material for: Prospective observational studies on nutrition intake and the incidence of cognitive impairment in middle-aged and older adults: A protocol for systematic review and meta-analysis
Source: PLoS One. 2023 Jun 29;18(6):e0287852. doi: 10.1371/journal.pone.0287852 (PMC10309612; doi:10.1371/journal.pone.0287852)
Supplement: S2 Table — (DOCX) [file pone.0287852.s002.docx]

Table 2 Characteristics of Study on nutrition intake for cognitive impairment

| Publication information | | Participants characteristics | | | | | | Exposure | | Study characteristics | | | | Outcomes | |
| --- | --- | --- | --- | --- | --- | --- | --- | --- | --- | --- | --- | --- | --- | --- | --- |
| first author | year | country | N | females (%) | Age  (‾χ±s) | Ethnicity | educational attainment | dietary pattern(type) | dietary pattern assessment tool | follow-up period | confounders | industry funding | diagnosis of cognitive impairment (assessment tool, threshold) | incidence | Result  (OR, RR, HR, β. SE, P_0_) |
|  |  |  |  |  |  |  |  |  |  |  |  |  |  |  |  |
